# Outcomes in Hirschsprung’s disease with coexisting learning disability

**DOI:** 10.1007/s00431-021-04129-5

**Published:** 2021-06-11

**Authors:** Joseph R. Davidson, Kristiina Kyrklund, Simon Eaton, Mikko P. Pakarinen, David Thompson, Simon C. Blackburn, Kate Cross, Paolo De Coppi, Joe Curry

**Affiliations:** 1 Department of Specialist Neonatal and Paediatric Surgery, Great Ormond Street Hospital for Children, London, UK; 2 Stem Cells and Regenerative Medicine Section, UCL Great Ormond Street Institute of Child Health, London, UK; 3 Division of Pediatric Surgery, New Children’s Hospital Helsinki and Helsinki University Hospital, Helsinki, Finland

**Keywords:** Hirschsprung’s, Learning disability, Down syndrome, Bowel function, Quality of life, Long-term outcomes

## Abstract

This study describes functional and health-related quality of life (HRQoL) outcomes in patients with Hirschsprung’s disease (HSCR) with associated learning disability or neurodevelopmental delay (LD), completing a core outcome set for HSCR. This was a cross-sectional study from a tertiary pediatric surgery center. Patients treated between 1977 and 2013 were prospectively contacted to complete an outcomes survey. Children under 12 and older patients with LD were assisted to complete these by a proxy. Bowel and urologic function were assessed (Rintala’s BFS and modified DanPSS) along with HRQoL (PedsQL/GIQLI/SF-36). Thirty-two patients with LD were compared to 186 patients with normal cognition. Patients with LD had 76% survival over the follow-up period, compared to 99% in the remainder of the cohort. Poor functional outcomes were common in the patients with LD, considerably higher than cognitively normal patients: with weekly issues withholding stool, soiling and fecal accidents in over half of patients surveyed (44–60%), and urinary incontinence in 46%. Use of permanent stoma was significantly higher (22% vs. 4%; p = 0.001). HRQoL was worse in domains of physical functioning in adults and children but not for social or emotional domains in adults. Subgroup analysis of patients with Down syndrome suggested similar functional results but better QoL. Multivariate analysis demonstrated a dramatically higher incidence of poor continence outcomes in patients with LD (adjusted OR 9.6 [4.0–23]).

*Conclusions*: We provide LD-specific outcomes showing inferior function but similar HRQoL to other patients with HSCR, this is much needed in the counselling of families of these children.

**Table:** 

**What is Known:** • *Hirschsprung’s disease is commonly associated with syndromes or other anomalies with resultant cognitive impairments.* • *The outcomes for these patients specifically have been poorly described in the literature.*	
**What is New:** • *Objective functional and quality of life surveys demonstrate significant differences from patients without cognitive impairment.* • *Patients with learning disability Patients with associated LD were almost ten times more likely to have an associated poor functional outcome, with very little impact on proxy-reported quality of life.*

**What is Known:**

• *Hirschsprung’s disease is commonly associated with syndromes or other anomalies with resultant cognitive impairments.*

• *The outcomes for these patients specifically have been poorly described in the literature.*

**What is New:**

• *Objective functional and quality of life surveys demonstrate significant differences from patients without cognitive impairment.*

• *Patients with learning disability Patients with associated LD were almost ten times more likely to have an associated poor functional outcome, with very little impact on proxy-reported quality of life.*

## Introduction

Hirschsprung’s disease (HSCR) is the outcome of a failed migration of neural crest cells to the anal canal, affecting a variable length segment of the distal intestine, with an incidence of approximately 1:5000. There are several known genetic or chromosomal syndromes with neurodevelopmental delay associated with HSCR, of which the most common is Down syndrome (Trisomy 21). Although inferior bowel functional outcomes are recognized associations of learning disability (LD) [1], these have not been specifically quantified in studies of long-term outcomes in HSCR to date. Herein, we describe a continuous cohort of adult and pediatric HSCR patients with LD with reference to cognitively normal HSCR patients [2], completing a core outcome set for these individuals [3]. These novel results provide a basis for counselling patients and families on the likely functional outcomes in this group of HSCR patients into adulthood.

## Methods

The records of all patients born between June 1977 and December 2013 who received surgical treatment for Hirschsprung’s (primarily or referral for redo pull-through) at our tertiary pediatric surgical center were reviewed; cases were identified through clinical coding for ICD-8,9,10 codes (ICD-7, i.e., pre-1977 were not possible to search). LD was defined as the presence of a syndrome with associated neurodevelopmental delay or patients with documented learning disability. The carers of all living HSCR patients residing in the United Kingdom were invited to complete a multi-domain questionnaire of functional outcomes and quality of life (QoL). Written informed consent to participate was obtained from patients and/or carers as appropriate; carers assisted children < 12 years of age and patients with LD in completing the survey. Results were reported according to the core outcome set (COS) domains defined by Allin et al. [3]. The breakdown of the COS and the corresponding instruments are listed in Table 1. The responses of patients with LD were compared to our previously collected data on cognitively normal HSCR patients from the same institution over the same time period [2]. Since the age ranges were wide and age at assessment is a known factor in continence outcomes in both healthy population and HSCR, we described bowel function and urinary function separately for patients ≥ 18 years and < 18 years of age. This study received National Research Ethics approval (17/LO/1692) and was conducted in accordance with the STROBE statement.

**Table 1:** Core outcome set for Hirschsprung’s (taken from NETS1HD study) [3]

COS domain	Instrument/method	
Survival		
1. Cause of death with cause specified	Retrospective review of medical records	Whole cohort (n = 428)
Hirschsprung’s related complications		
2. Unplanned reoperation 3. Need for permanent stoma	Retrospective review of medical records and prospective enquiry	Review of notes and response to cross-sectional prospective follow-up (n = 218)
4. Hirschsprung-associated enterocolitis (HAEC)	Review of records (clinician recorded HAEC) and prospective enquiry (recurrent or recent HAEC)	
Bowel function		
5. Long-term fecal incontinence 6. Long-term voluntary bowel movements, need for enemas 7. Objective score of bowel function	Prospective follow-up, Bowel Function Score [4]	Respondents to cross-sectional prospective follow-up (n = 218)
Urological function		
8. Long-term urinary incontinence	Prospective follow-up, modified DanPSS [5]	
Psychological impact + quality of life		
9. Long-term psychological stress 10. Objective score of quality of life	Prospective follow-up; items within bowel and urologic function, PedsQL, SF-36, GIQLI	

Results were compared with appropriate categorical and non-parametric testing; p < 0.05 was considered statistically significant. All statistically significant results were accompanied with a measure of difference, partial eta-squared (η^2^) for continuous variables, or for categorical variables: odd’s ratio + 95% confidence interval. The factors associated with poor functional outcome were explored using logistic regression. Covariates were selected based upon prior exploration in a study of cognitively normal patients [2] and included age, sex, redo pull-through, and segment length: rectosigmoid vs. extended segment (long segment (LS) and total colonic aganglionosis +/− small bowel involvement (TCA)). An additional variable of constipation (requiring laxatives, enema, or ACE) was added to the multivariate analysis for frequent urinary incontinence (weekly or daily symptoms). We performed further logistic regression for clinically significant cut-offs of PedsQL and GIQLI (SF-36 data is typically analyzed as either subdomains only or as component scores for which a normative dataset is required); covariates were age, sex, redo pull-through, segment length, poor bowel function, and daily urinary incontinence.

A subgroup analysis was performed for patients with Down syndrome, in order to demonstrate that the results and conclusions might be applicable to these patients specifically, since they make up such a large proportion of patients with HSCR.

## Results

### Demographics, clinical management, and outcomes

**Fig. 1:**
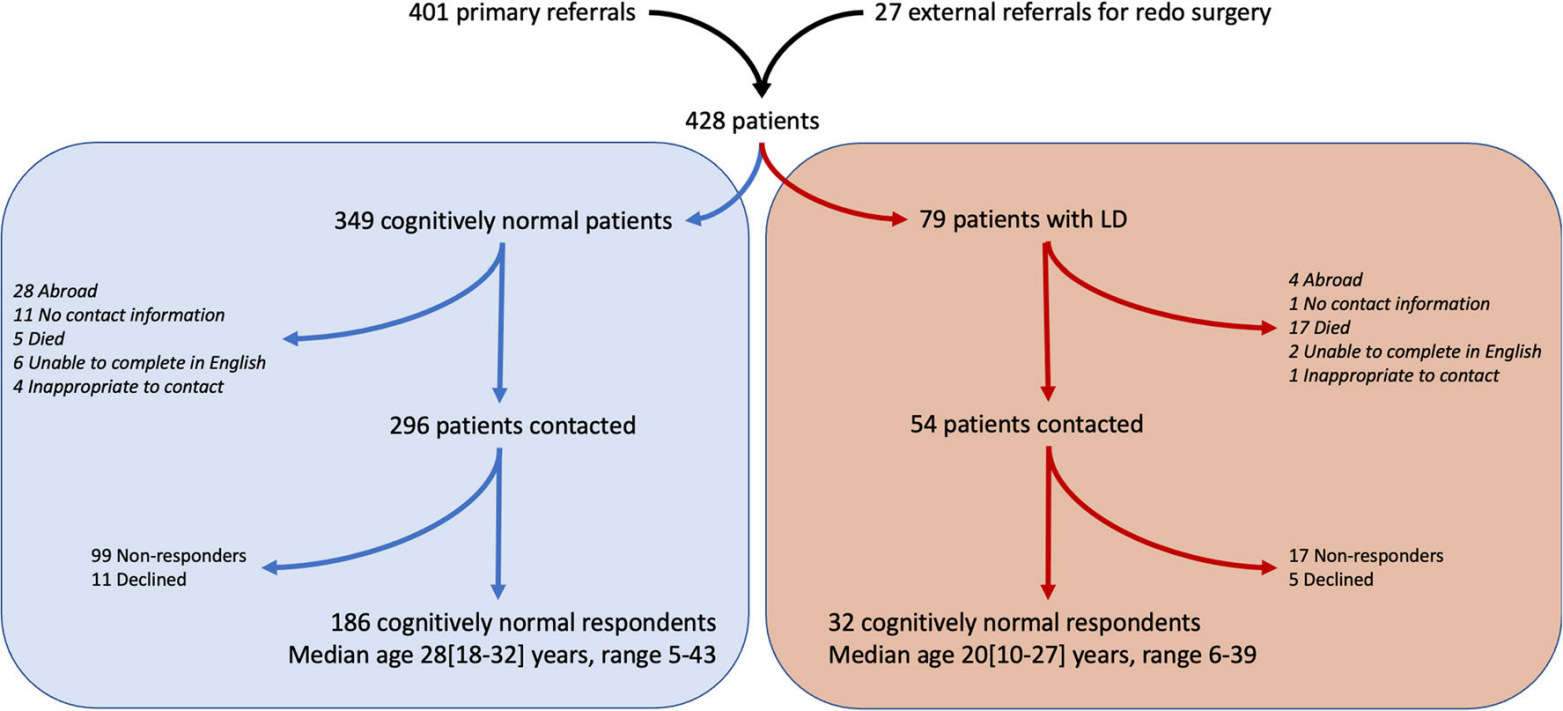
Study inclusion flowchart

Of 401 patients treated primarily for HSCR and 27 who had undergone redo surgery, 100 had an additional major anomaly or syndrome; of this group, 79 (20%) had LD (Fig. 1), of whom 54 were alive and eligible for inclusion; 32/54 (59%) eligible LD patients returned the outcomes questionnaires vs. 186/278 (67%) cognitively normal patients (p = 0.28). Down syndrome was the most common associated condition, with an overall incidence of 9.5% of HSCR patients (n = 18; 50% of LD patients). There was a higher proportion of patients with LD who had extended segment disease (39%: 20% LS, 19% TCA) compared to those without LD (25% extended segment: 14% LS 11% TCA; p = 0.015). Initially, pull-through surgery was performed in 71 LD patients (90%), the remaining 8 (10%) underwent definitive management with stoma formation due to complex and life-limiting associated congenital anomalies and had passed away at the time of study. This proportion of patients with definitive stoma formation was significantly higher than among cognitively normal patients (n = 3/349, all with TCA, p = 0.0001).

### Outcomes assessment

Thirty-two patients with LD and 186 cognitively normal patients were included within the outcomes assessment. Study inclusion flowchart and dropout analysis is shown in Fig. 1 and Table 2; there were no differences between those eligible patients with LD who took part (n = 32) and those who did not respond or declined to take part (n = 18); the dropout analysis for the control group of cognitively normal patients has already been published [2]; however as is illustrated in Tables 2 and 3, most patients in the cohort underwent a Duhamel pull-through, and most had rectosigmoid disease (although this proportion was lower in patients with LD). Incomplete datasets (5/32) were included but accounted in the denominator of the relevant analyses. Comparing to the patients without learning disability, there were no significant differences in age or operative management between cohorts. We noted there was slightly higher proportion both of female patients and patients with extended segment disease in the LD groups as would be expected; there were more patients with Down syndrome in the adult group.

**Table 2:** Dropout analysis for LD patients

	Respondents (n = 32)	Non-respondents (n = 22)	P
Age, y (median [IQR])	20 [10–27]	23 [13–30]	0.52
Sex, m:f	21:11	16:6	0.77
Down syndrome, n (%)	18 (56)	14 (64)	0.79
Rectosigmoid segment, n (%)	20 (63)	18 (82)	0.22
Duhamel pull-through, n (%)	31 (97)	18 (82)	0.15

**Table 3:** Comparison of patients with LD to cognitively normal patients with HSCR

Under 18	LD (n = 15)	Cognitively normal (n = 47)	P
Age, y (median [IQR])	9 [7–14]	11 [7–14]	0.57
Sex, m:f	10:5 (2:1)	37:10 (3.7:1)	0.34
Down syndrome, n (%)*	7 (47)	-	-
Rectosigmoid segment, n (%)	10 (66)	37 (79)	0.30
Duhamel pull-through, n (%)	15 (100)	46 (98)	0.72
Adults	LD (n = 17)	Cognitively normal (n = 139)	P
Age, y (median [IQR])	27 [23.5–31.5]	29 [25–34]	0.42
Sex, m:f	11:6 (1.8:1)	98:41 (2.4:1)	0.62
Down syndrome, n (%)*	11 (65)	-	-
Rectosigmoid segment, n (%)	10 (59)	104 (75)	0.26
Duhamel pull-through, n (%)	16 (94)	108 (78)	0.20

*Other conditions included Mowat-Wilson syndrome and Goldberg-Schpritzen syndrome, along with various chromosomal anomalies and global developmental delay with or without autism

## Core outcome set

### Survival

**Fig. 2:**
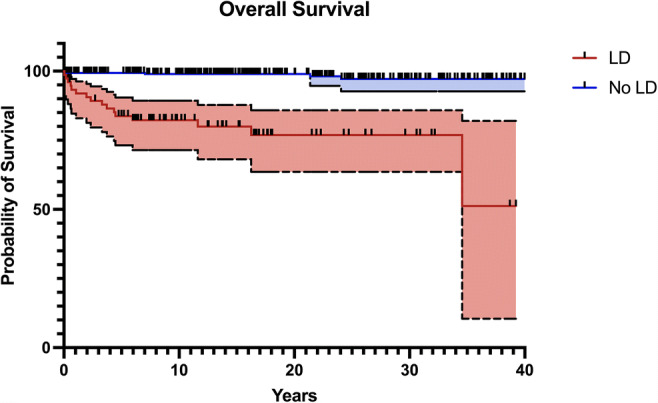
Kaplan-Meier survival curves for HSCR patients with and without LD. Logrank test performed, hazard ratio 15.68 [5.0–49.0]; p < 0.0001

The overall survival (as of the time of the study) in the 79 patients with LD was 76% (vs. 99% in no LD; logrank HR 15.68 [5.0–49.0]; p < 0.0001, Fig. 2); cause of death in these patients was uniformly related to major syndromic anomalies (severe congenital cardiac, metabolic, or neurodegenerative disease in the majority) and not to Hirschsprung’s; contrastingly, 3 of the 5 deaths in patients without associated anomaly were related to neonatal sepsis (prior to pull-through).

### Complications of Hirschsprung’s and pull-through surgery

#### Surgical complications

After primary pull-through, 78% (25/32) of LD respondents remain with intestinal continuity and 22% (n = 7/32) have undergone subsequent stoma formation due to poor bowel function and/or incontinence (vs. 7/186 4% of cognitively normal patients; OR 7.16 [2.3–22.1] p = 0.001), including two patients in whom redo pull-through was first attempted to improve symptoms. A third patient with LD also underwent redo surgery but continues to have daily fecal accidents and soiling. Overall, redo pull-through was performed in 3/32 (9%) patients with LD (vs. 27/159 (17%); p = 0.44), although it is important to note that the cohort contained patients who were referred from external centers specifically for redo surgery. We observed similar incidence of complications of anastomotic leak (0/32 (0%) vs. 13/186 (7%); p = 0.12) and anastomotic stricture/spur requiring a procedure (5/32 (16%) vs. 27/186 (15%); p = 0.87).

#### Hirschsprung’s associated enterocolitis

Based on clinical records, Hirschsprung’s associated enterocolitis (HAEC) was more prevalent: 13/32 (40%) vs. 37/186 (20%), p = 0.021, and patients with LD were more likely to have had an episode of HAEC in the preceding year (4/32 (13%) vs. 6/186 (3%), p = 0.043). Learning disability was added as a covariate to linear regression analysis and a significant correlation between lower BFS and recent episodes of HAEC (p = 0.035) was observed; with LD also a highly significant correlate (p < 0.001).

### Bowel outcomes

#### Long-term continence, control, and need for enemas

**Fig. 3:**
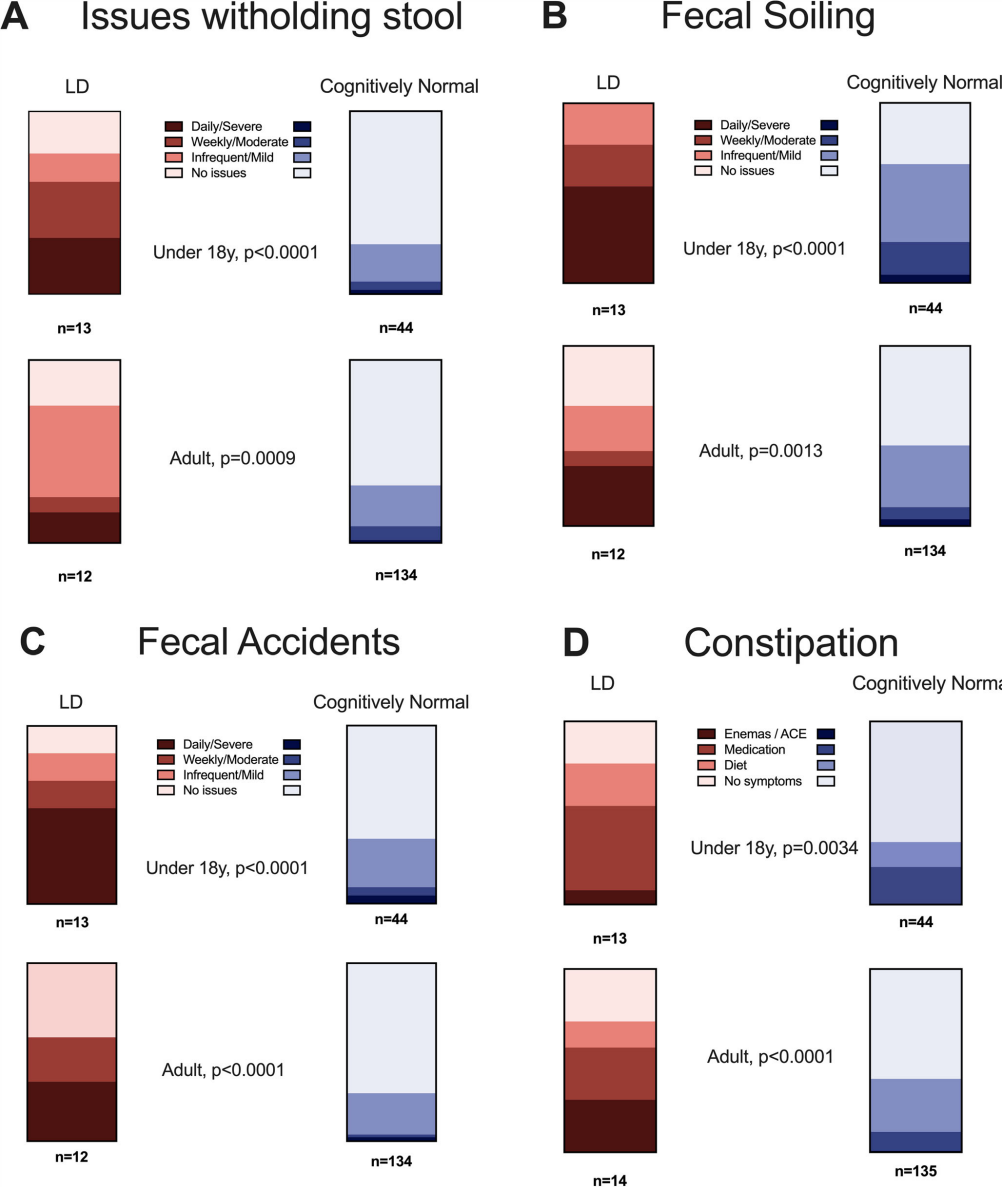
BFS items (**A**: issues witholding stool, **B**: fecal soiling, **C**: accidents, **D**: constipation) reported in under 18 and over 18 years; comparison with chi-square test for trend. NB constipation patients also include those with ACE irrigation (n = 3, who are not formally assessed with BFS otherwise)

In addition to the 22% of patients (7/32) with stoma referred to in “Surgical complications” section above, we explored current bowel function and management using the BFS. There was significantly higher frequency of symptoms regarding withholding stool, fecal soiling, accidents, and constipation in both age groups (Fig. 3). Daily soiling (U18: 54% vs. 5%; adult 33% vs. 2%) and daily fecal accidents (54% vs. 5%; adult 33% vs. 2%) were common in patients with LD. Rectal enemas were used in 3/25 LD patients, and a further 2 adult LD patients used an ACE irrigation daily.

#### Objective score of bowel function

**Fig. 4:**
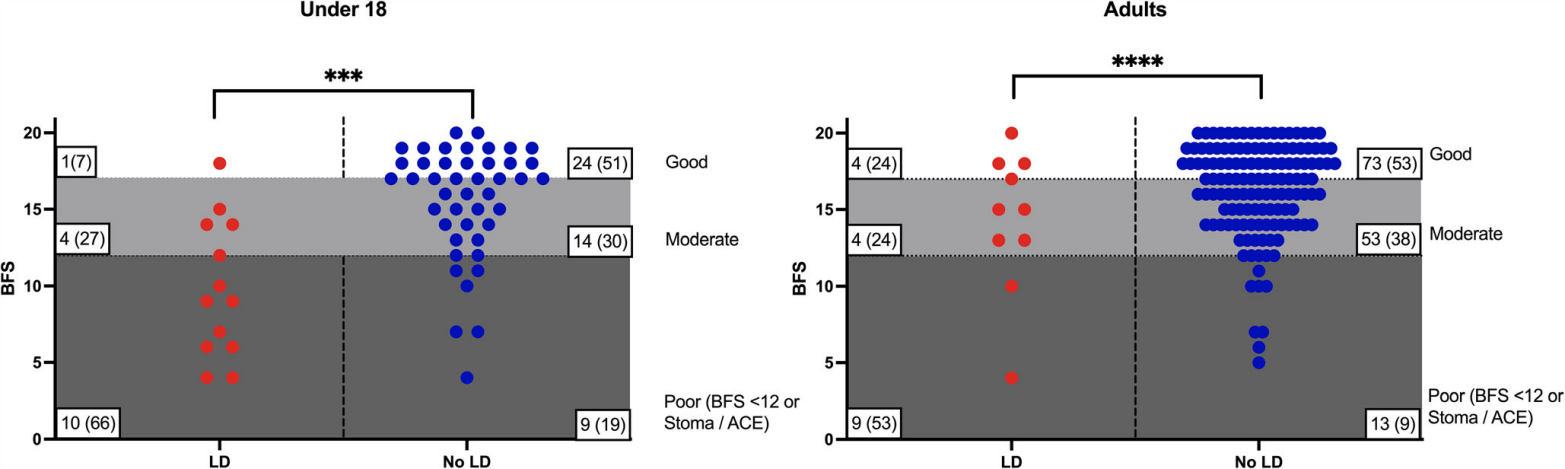
Scatter plot of patients with LD and cognitively normal patients demonstrating overall BFS. Sections of the graph drawn represent good, moderate, and poor outcomes with differences between two groups assessed with chi-square test for trend (***p < 0.001, ****p < 0.0001). Numbers within each section represent n (%). Numbers with poor outcome include patients with stoma/ACE who are not charted as they are not scored with BFS

By BFS, the overall bowel functional outcome was inferior among LD patients without ACE or stoma versus cognitively normal patients, as shown in Fig. 4. LD was a significant factor on multivariate analysis for the outcome of poor bowel outcome, defined as BFS < 12 or need for permanent stoma or ACE (OR 9.6 [4.0–23], p < 0.001).

### Urologic outcomes

Urinary incontinence was more common in patients with LD: weekly/daily urinary incontinence (12/32 (38%) vs. 20/185 (11%), p < 0.001). Urinary tract infection (12/30 (40%) vs. 49/185 (26%)) and frequent lower urinary tract symptoms (11/27 (41%) vs. 48/185 (26%)) were also noted more prevalent in patients with LD although these were not statistically significant (p > 0.1 for both). On multivariate analysis, LD was a significant predictor of frequent urinary incontinence (OR 3.77 [1.5–9.6]; p = 0.006) along with presence of constipation managed with laxatives, enema, or ACE (OR 2.81 [1.1–7.4]; p = 0.035).

### Psychological and quality of life outcomes

In the 203 patients assessed with BFS, bowel function was responsible for severe psychological impact in few patients (7% overall), and social impact was noted in LD patients as frequently as cognitively normal patients (U18, 3/13 (23%) vs. 7/44 (16%); p = 0.41. Adults, 3/12 (25%) vs. 24/134 (18%); p = 0.47). Importantly, patients with stoma were not assessed by this instrument.

#### PedsQL

Children with LD (n = 15) were assessed on the PedsQL and compared to their peers without LD (n = 46). Overall age-adjusted impaired scores (defined by Huang et al. [4]) were noted in 87% (13/15) of children with LD compared to 42% (20/46) cognitively normal children (OR 8.5 [1.7–42]; p = 0.006). Exploring the subdomains, there were significant differences with large effect sizes in physical (η^2^ = 0.19; p = 0.001) and social domains (η^2^ = 0.35; p < 0.001), and moderate effect sizes in those for emotional (η^2^ = 0.10; p = 0.016) and schooling domains (η^2^ = 0.11; p = 0.011). Logistic regression analysis demonstrated that an impaired PedsQL overall score was independently associated with poor bowel functional outcome (OR 5.1 [1.2–22]; p = 0.029) but the presence of learning disability did not reach statistical significance (OR 4.6 [0.84–26]; p = 0.078), nor did patient sex or extended segment disease.

#### SF-36 + GIQLI

Adults with LD (n = 17) were assessed by both SF-36 and GIQLI via a proxy and compared to cognitively normal patients (n = 139). Examining the individual SF-36 domains, there was a difference observed in physical functioning which was lower in LD patients (η^2^ = 0.1; p < 0.001) but not in any other domain assessed. GIQLI scores were not noted to be different on comparison of overall scores (118 [106–128] vs. 121 [104–131]; p = 0.53, Mann-Whitney test) and a similar proportion of patients met the cut-off of GIQLI ≤ 105 (4/17 (24%) vs. 35/139 (25%); p = 0.88). On multivariate analysis for the outcome of impaired GIQLI score, LD was a significant predictor of a higher GIQLI score (aOR 5.20 [1.06–25.5]; p = 0.042), along with poor bowel function (aOR 11.21 [3.02–41.7]; p < 0.001), extended segment (aOR 3.85 [1.62–9.15]; p = 0.002), and female sex (aOR 2.69 [1.14–6.37]; p = 0.024). Interestingly, cognitively normal patients living with a stoma (n = 4 vs. 135 without) scored significantly lower in the GIQLI social subdomain (Bonferroni-corrected p-value = 0.032) but no difference in any other part of the score. Comparatively among patients with LD, there was no difference in GIQLI subdomain scores between the patients with stoma and those in intestinal continuity (in fact the median scores for social and emotional functioning were higher).

### Effect of Down syndrome

Given the marked heterogeneity of the cohort in terms of underlying conditions, we compared those patients with Down syndrome (n = 18) to other LD patients without Down syndrome (n = 14). Functional outcomes were somewhat better in patients with Down syndrome; however, these differences were not statistically significant: with incidence of poor bowel outcome (9/18 (50%) vs. 10/14 (71%); p = 0.29) and frequent (weekly) urinary incontinence (4/18 (22%) vs. 8/14 (57%); p = 0.07). There were no differences in PedsQL between those with (n = 7) and those without Down syndrome (n = 8; p = 0.9). Overall GIQLI score was similar in patients with Down syndrome and those without (120 [109–129] vs. 111 [62–125]; p = 0.3); scores were higher across all subdomains but not statistically significantly so. However on exploration of SF-36 domains, there were large-sized differences with scores in patients with Down syndrome that suggested better physical functioning (η^2^ = 0.31; p = 0.02) and less physical role limitation (η^2^ = 0.43; p = 0.005).

## Discussion

Considering that syndromic conditions with LD are a significant feature of HSCR, the outcomes in this subset of patients have historically been poorly described [1], with reports often lacking formal validated scoring. Similarly, patient numbers in retrospective cohorts have limited statistical power to detect clinically significant differences [5]. Our results suggest that patients with LD are nearly 10 times more likely to have a poor functional outcome compared to cognitively normal patients according to a multivariate model controlling for age (which we have shown to be factors associated with poorer outcome in the cognitively normal patient population [2]). We also found a high incidence of moderate/severe problems withholding defecation, fecal accidents, soiling, constipation, and HAEC. This aligns with other published studies where objective functional measures have been reported in patients with HSCR [6, 7].

Our previous work examining the cognitively normal patients within this group identified a complex inter-relationship of functional status and health-related quality of life [2], and exploration of this in patients with LD reveals further insights. While bowel and urinary functional outcomes were markedly impacted, there appeared to be less impact of this on HRQoL in LD. There were significant physical subdomain differences in both children and adults as expected, given the issues associated with the complex associated diagnoses. However, domains of emotional function seemed to be less impacted in children, and not at all in adults with either of the instruments used. It was surprising that the presence of a learning disability was correlated with improved GIQLI score on the multivariate model. This work suggests that fecal incontinence after reconstructive surgery is common and is likely to be more challenging to manage than among patients without LD. Understanding the differences in the likely functional outlook after reconstructive surgery and that stoma formation may not be detrimental to QoL in selected patients is important for counselling families regarding the treatment options and for directing management based on individual clinical circumstances [8].

The apparent impact on pediatric but not adult QoL scoring in LD should be interpreted with caution, as the study is cross-sectional and therefore survivorship bias is possible — and certainly might be predicted given the increased proportion of patients with Down syndrome whereas the younger half of the LD cohort had certain diagnoses known to be life-limiting. Parents may also sometimes overestimate the effect of symptoms on a child’s QoL [9] and parents of children with LD in particular may expect a more significant impact on QoL [10]. The limited number of HSCR patients with LD in this study was an expected limitation of this study of a rare disease; however, the size of the cohort is significant considering the relative paucity of literature available for this patient group. Limitations concerning the validity of findings in any proxy-based assessment in LD and in young children are also clear.

Although presence of LD was a significant factor on univariate analysis for urinary incontinence, it did not remain so after the addition of poor bowel outcome into the multivariate model. This reflects findings from a similar study of urologic outcomes (specifically in Down syndrome with HSCR) [11]. The heterogeneity of syndromes prompted a subgroup analysis of the most common associated condition, Down syndrome. We found these patients’ functional outcomes to be comparable with the overall LD cohort; however, there were some but with some positive differences observed in the QoL metrics in adults. Characteristic features of Down syndrome include a social nature and cheerful disposition, and families report lower levels of stress and a more positive outlook than in other forms of LD, which may explain these findings, which, it must be remembered, were based upon proxy-reported outcomes [12]. It is certainly a limitation of this study that an estimation of severity of intellectual impairment was not possible based on medical records and survey-based assessment. Severity of intellectual impairment is known to have a considerable impact on continence outcomes [13], and further work in this area ought attempt to quantify this in patients with HSCR. An instrument such as the Learning Disability Screening Questionnaire (LDSQ), while easier to process than formal IQ testing, does still require face-to-face contact and hence was not feasible within the remit of our current study [14, 15].

It is a further limitation of our study that we are not able to compare these data to those from LD patients without HSCR. Studies of wider groups of patients with intellectual impairment have been performed. An observational study in a cohort of persons with Down syndrome estimated urinary incontinence of 17% and fecal incontinence of 14% overall — with a delayed attainment of control (around 12 years) and a subsequent regression in older adults (> 30 years) [16]; clearly, the rates of fecal incontinence we described are considerably higher. The focus of our work has been to focus on the relative outcomes between patients with HSCR with and without associated LD: objective evidence to this end is important to gather, not in the least because of the high incidence of Down syndrome in the HSCR population. Furthermore, long-term prognosis for continence and eventual use of permanent stoma in patients is a key concern for parents; therefore, we envisage this study will provide both mid- and long-term information for families, which is currently needed.

## Abbreviations

ACE: Antegrade continence enema (i.e., Malone appendicostomy or cecostomy)
BFS: Bowel Function Score
COS: Core outcome set
DanPSS: Danish Prostatic Symptoms Score
GIQLI: Gastrointestinal Quality of Life Index
HAEC: Hirschsprung’s associated enterocolitis
HR: Hazard ratio
HSCR: Hirschsprung’s
ICD: International Statistical Classification of Diseases and Related Health Problems
LD: Learning disability
LS: Long-segment Hirschsprung’s (i.e., aganglionosis extending past the sigmoid colon)
OR: Odds ratio
PedsQL: Pediatric quality of life (here version 4.0)
QoL: Quality of life
SF-36: Short-Form 36
TCA: Total colonic aganglionosis
